# Analysis and Optimization of Pulse Dynamics for Magnetic Stimulation

**DOI:** 10.1371/journal.pone.0055771

**Published:** 2013-03-01

**Authors:** Stefan M. Goetz, Cong Nam Truong, Manuel G. Gerhofer, Angel V. Peterchev, Hans-Georg Herzog, Thomas Weyh

**Affiliations:** 1 Technische Universität München, Institute of Energy Conversion, Munich, Germany; 2 Duke University, Department of Psychiatry and Behavioral Sciences, Department of Biomedical Engineering, and Department of Electrical and Computer Engineering, Durham, North Carolina, United States of America; Glasgow University, United Kingdom

## Abstract

Magnetic stimulation is a standard tool in brain research and has found important clinical applications in neurology, psychiatry, and rehabilitation. Whereas coil designs and the spatial field properties have been intensively studied in the literature, the temporal dynamics of the field has received less attention. Typically, the magnetic field waveform is determined by available device circuit topologies rather than by consideration of what is optimal for neural stimulation. This paper analyzes and optimizes the waveform dynamics using a nonlinear model of a mammalian axon. The optimization objective was to minimize the pulse energy loss. The energy loss drives power consumption and heating, which are the dominating limitations of magnetic stimulation. The optimization approach is based on a hybrid global-local method. Different coordinate systems for describing the continuous waveforms in a limited parameter space are defined for numerical stability. The optimization results suggest that there are waveforms with substantially higher efficiency than that of traditional pulse shapes. One class of optimal pulses is analyzed further. Although the coil voltage profile of these waveforms is almost rectangular, the corresponding current shape presents distinctive characteristics, such as a slow low-amplitude first phase which precedes the main pulse and reduces the losses. Representatives of this class of waveforms corresponding to different maximum voltages are linked by a nonlinear transformation. The main phase, however, scales with time only. As with conventional magnetic stimulation pulses, briefer pulses result in lower energy loss but require higher coil voltage than longer pulses.

## Introduction

### Background

Magnetic stimulation is a standard tool for noninvasive activation of neurons. It can be applied to the brain, where it is known as transcranial magnetic stimulation (TMS) [1], and in the periphery, e.g., for neuromuscular stimulation [2]. Based on the principle of electromagnetic induction, this technology is not impeded by low-conductivity tissue, such as bone, causes little distress, and can be relatively well focused. In both central and peripheral magnetic stimulation, the key advantage is its tolerability.

Compared to other stimulation methods, however, magnetic stimulation is extremely energy inefficient. The electrical pulse energy transferred to the stimulation target is less than one percent [3], [4]. Although a substantial fraction of the field energy can be retrieved and used in a subsequent pulse, the high pulse currents in the stimulator circuit in the range of kiloamperes lead to significant ohmic losses. In repetitive stimulation protocols, the high losses heat the coil and limit either the treatment duration or the pulse parameters, such as repetition rate and strength. Neuromuscular magnetic stimulation, which targets neurons in order to activate muscles, is in this context very critical [2]. The high powers limit sessions with standard equipment to a few minutes–which is insufficient for treating, for instance, atrophy–and compromise a successful clinical application.

Furthermore, driving the high currents necessitates voltages up to 4000 V which requires reliable high voltage insulation. Mobile devices with battery-powered pulse sources have been proposed but are at present not practical for repetitive protocols due to the high power consumption.

While the efficiency of magnetic stimulation in the spatial domain has been studied extensively with appropriate coil designs [5]–[9], including the incorporation of magnetic materials [10], [11], the role of waveforms in this aspect has received less attention.

In the temporal domain, the induction process, wherein an alternating coil current induces an electric field, is approximately linear. However, the neuron dynamics are complex and nonlinear, resulting in a nontrivial relationship between the pulse waveform and the neural response. Nevertheless, the conventional waveforms are limited by the relatively simple and inflexible stimulator circuit topologies rather than derived from consideration of what is optimal from the perspective of a neuron.

Presently, there are two waveforms–monophasic and biphasic–commonly found in magnetic stimulation devices. Standard monophasic stimulators generate an overdamped sine current pulse and dissipate the whole pulse energy as heat [12]. In biphasic (and multiphasic) devices, the oscillation is underdamped, which leads to an almost sinusoidal waveform, and can recover a substantial amount of the pulse energy [8], [13]–[16]. Recent stimulator designs enable the generation of approximately triangular current pulses, corresponding to near-rectangular electric field pulses [17]. Whereas such pulses seem to be more efficient than conventional sinusoidal pulses [17], [18], they have not been shown to be optimal.

The question of the optimal pulse shape for neural activation has been studied in more detail in electrical stimulation. An analytic variational approach with a linear first-order neuron model, also known as leaky integrate-and-fire model [19], resulted in the so-called rising-exponential waveform for a minimum pulse energy [20]. This work was reproduced later [21]. However, the results are compatible neither with more sophisticated neuron models [22.nor with experiments [23].

In contrast to linear dynamics, nonlinear models cannot be inverted easily and chaotic behavior of the corresponding differential equations renders numerical handling problematic. Therefore, many parameter studies in models as well as in experiments were instead performed with specific predefined waveforms [23]–[28]. A general, largely unbiased optimization of the waveform for electrical stimulation minimized the ohmic losses of monophasic electrical pulses [29]. The reported optima are better described by a Gaussian curve than by the rising exponential function predicted by the linear neuron model.

For magnetic stimulation, there are few studies of different waveforms with experiments [15, 16, 18, 30.or numerical models [31]–[34]. Likewise, these studies explored limited sets of predefined pulse shapes. Thus, the question of the optimality of magnetic stimulation waveforms remains largely unanswered. In this paper, we optimize the waveform for magnetic stimulation for minimum energy loss, which is equivalent to coil heating, using a nonlinear neuron model and without constraining the pulses shape unnecessarily.

### Objectives and Method Overview

The magnetic stimulation pulse waveform could be optimized with respect to several aspects. In this work, we minimize the energy loss during a pulse, which affects the required power supply and causes coil heating. The device power consumption and heating of the stimulation coil are both approximately proportional to the energy loss and limit the maximum duration of a stimulation session as well as the pulse repetition rate [35]. The approach avoids any unnecessary constraints, such as limitations of existing stimulation technology, which provides only a limited set of waveforms and is furthermore not able to generate all of these existing pulse shapes with energy recovery from the coil.

The dominating mechanism of energy loss is Joule heating originating from the intrinsic resistance of all electrical components. For coil current 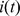, the energy loss of a pulse is
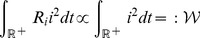
(1)for any value of the intrinsic series resistance 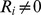. The second integral in Equation (1) forms the objective functional 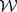, referred to as energy loss, which is used for further analysis and optimization.

Only two constraints are imposed on the optimization problem. First, the coil current has to successfully elicit a neuron response. Second, the coil voltage 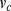 should not exceed a limit 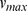, both in positive and negative polarity, 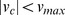. The latter constraint results from the observation that the coil energy and the ohmic loss at the excitation threshold fall with shorter pulse durations for classical waveforms, while the required coil voltage increases [4], [32]. Consequently, optimization that does not impose a maximum voltage constraint would result in divergence of the voltage. Furthermore, the voltage constraint is a practical design limit in magnetic stimulation devices motivated by the limitations of semiconductor devices, insulation, and related patient safety considerations. Voltage 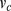 refers to the voltage of the inductance of the coil only. This voltage does therefore not depend on the resistance of the coil or other components, such as cables and connectors.

It should be noted again that this optimization formulation addresses only the pulse waveform and deliberately disregards specific device circuit implementations. Consequently, additional loss mechanisms that are circuit-dependent and can in principle be avoided or minimized, such as forward bias voltages of semiconductor p-n junctions and capacitive switching losses, are not considered in the optimization. In addition, only temporal aspects of the stimulation system are addressed. The coil design and the spatial characteristics of the induced field are not considered, except for the coil’s inductive and resistive properties which pertain to the time domain. This approach relies on the good separability of space and time in neuronal stimulation and is commonly deployed to decouple the spatial and the temporal domain [11], [36].

In summary, the optimization approach identifies a function 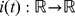 representing the coil current such that the loss per pulse (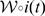) is minimal on condition that the pulse successfully leads to an action potential and the coil voltage required for generating this current in a coil with linear inductance is below a predefined limit.

The functions 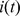 are parametrized so that numerical handling becomes possible. This is based on spline curves and Fourier series whose degrees of freedom are handed over to an optimization algorithm. For spline curves, these degrees of freedom are mathematically equivalent to the location of anchor points; for Fourier series they involve the amplitude and phase information of the frequency content. This parametrization is stable and general so that in principle any waveform, even (bandwidth-limited) noise, can be approximated for a sufficiently high number of degrees of freedom. In this study, the maximum duration of the waveforms 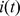 was limited to three milliseconds, since in pilot runs longer pulses had not been competitive. The waveform parameters are passed to a hybrid global-local optimization algorithm that provides both fast local refinement and global convergence.

A peripheral motor axon was used in the neuron model for the following reasons. First, in functional peripheral stimulation, such as neuromuscular stimulation, which targets motoric innervations [37], the problem of high power consumption and heating is most pressing and limits the application of the technology, for instance, in rehabilitation [2]. Second, motor axons are next of kin to axons originating from cortical pyramidal neurons with respect to channel expression [38, 39.but notably better studied than the heterogeneous situation in brain axons [40]–[42]. Consequently, peripheral motor axons are routinely used as the basis for the simulation of various neuron types, for example, by incorporating them as a compartment into cerebral neuron models [22], [23], [29], [43]–[47]. Since at present there is limited understanding of the specific neurons and activation sites recruited in TMS [48], [49], it is not possible to justify specific neuron model parameters for cortical stimulation. We will show that the conditions for energy optima in magnetic stimulation are relatively insensitive to the neuron model characteristics and hence are qualitatively general. Therefore, our results are likely applicable to various neuronal stimulation targets, including cortical neurons. Details of the models, the optimization framework and the numerical implementation are given in the methods section.

## Results

### Preliminary Exploration of the Waveform Space

**Figure 1:**
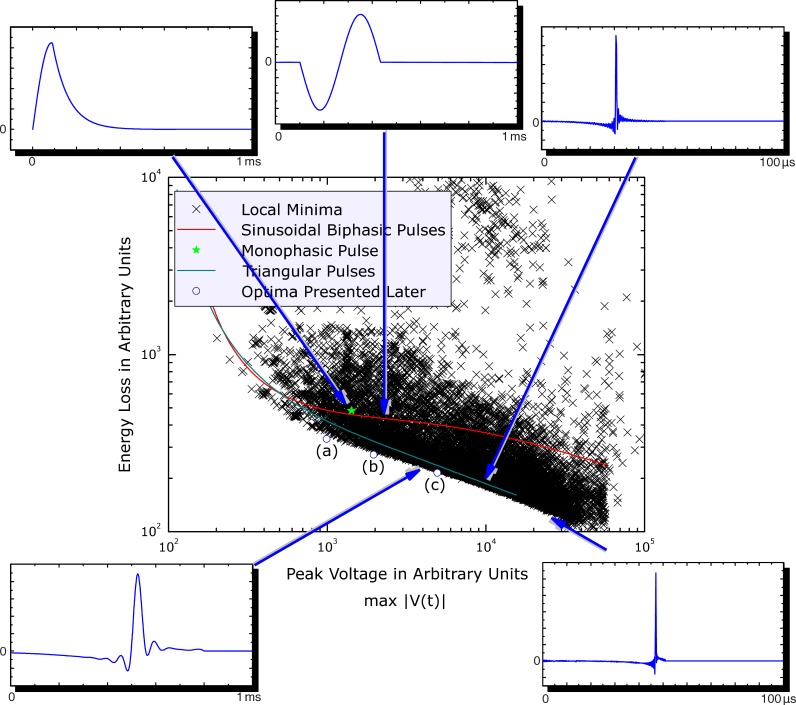
Heating loss versus maximum coil voltage for various waveforms. The local minima from the pre-evaluation are denoted by crosses. Sinusoidal biphasic and symmetric triangular current (i.e., rectangular voltage) pulses are plotted with red and cyan lines, respectively. The voltage and energy loss axes are in arbitrary units. The insets show some examples of current shapes. The lower-case letters refer to solutions which are shown in Figure 3.

A pre-evaluation of the optimal current waveforms was performed using the optimization method as an exploration tool in order to provide an approximate map of the solution space. It also acted as a test for the stability of the algorithms. The results are presented in Figure 1 which shows the energy loss as a function of the maximum coil voltage at the action potential threshold. Every cross in the graph represents a local minimum that corresponds to a certain waveform. In addition, data for standard biphasic sinusoidal (red line) and symmetric triangular current pulses (cyan line, corresponding to symmetric rectangular voltage pulses) were added from a parametric study for a frequency range from 500 Hz to 50 kHz. Moreover, the waveform of a commercial monophasic device (Magstim 200, Magstim Co., Whitland, Wales) was added (green star). In all cases, the current direction associated with the lowest threshold is represented.

In Figure 1, the voltage on the x-axis is in relative or arbitrary units and not calibrated to a specific threshold, but it lies in the range of real devices. Calibration of the x-axis can be easily accomplished based on available threshold data for conventional pulses. For example, the sinusoidal monophasic pulse generated by the commercial device Magstim 200 corresponds to an average motor threshold of about 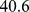
% of the maximum device amplitude which is 2800 V [50]. Thus, the typical threshold voltage for the sinusoidal monophasic pulse is 1137 V, which is commensurate with the voltage corresponding to the monophasic pulse in Figure 1.

Notably, the conventional monophasic waveform is insignificantly less efficient than conventional biphasic pulses, despite its relatively long current tail. Thus, conventional monophasic devices are substantially less efficient than biphasic devices not because of the intrinsic waveform properties, but rather because of the inefficient electrical circuit that shapes the pulse with intentional damping by a resistor that dissipates the pulse energy in the form of heat. This observation might call for better circuit topologies since monophasic pulses may be preferable in some applications due to their higher stimulation selectivity and stronger neuromodulatory effect [14], [51], motivating the development of devices that can efficiently generate monophasic and other pulse shapes that generate predominantly unidirectional electric field.

Figure 1 demonstrates that for sinusoidal and triangular current pulses the losses can be reduced with higher coil voltages. This confirms published observations for conventional harmonic pulses [4], [8], [32], also implied in [52]–[54], and for asymmetric near-triangular current pulses [17], [55]. For sinusoidal and triangular current pulses, scaling with voltage is associated with simple temporal dilation or compression corresponding to conventional strength-duration curves for the neural activation threshold [56]. The energy loss advantage of brief pulses is sometimes used for reducing the heating in high-power applications, e.g., in rehabilitation [2]. Triangular waveforms as well as all pulse shapes that form the lower edge of the scatter of local minima (see Figure 1) have a steeper slope of loss versus voltage than sinusoidal pulses, indicating that the gain in efficiency at higher voltages is larger for the former than for the latter.

This lower edge of the scatter is smooth and appears to represent the same class of stimuli. For very high device voltages, the current waveforms degenerate to pulses resembling a Dirac delta.

### Refined Optimization: Common Features of Optimal Waveforms

**Figure 2:**
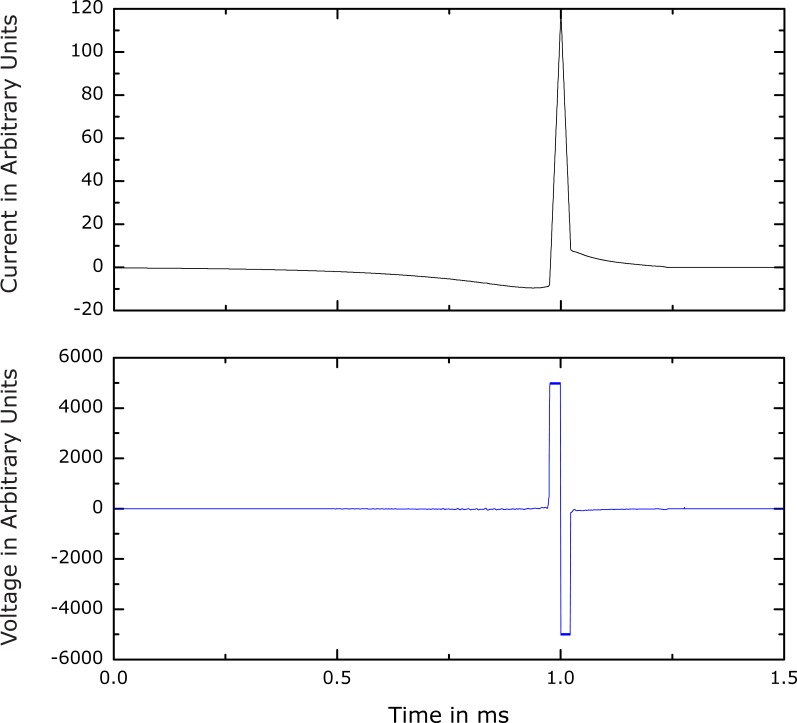
Current and voltage waveform. These plots correspond to waveform (c) in Figure 1. As defined in Section *Objectives and Method Overview*, the voltage refers to the inductive component of the coil and is directly proportional to the derivative of the current. Both voltage and current are scaled in arbitrary units. The absolute values of the current and voltage waveforms depend on many additional parameters such as coil design, inductance, distance to target, etc. The rise time of the main phase of the current pulse is 23 µs. The maximum derivative within the first phase is about 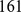 times lower than the slopes of the second phase. Despite the low amplitude, the long duration results in an area under the initial current phase that is higher than the area under the second phase by approximately 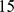
%.

**Figure 3:**
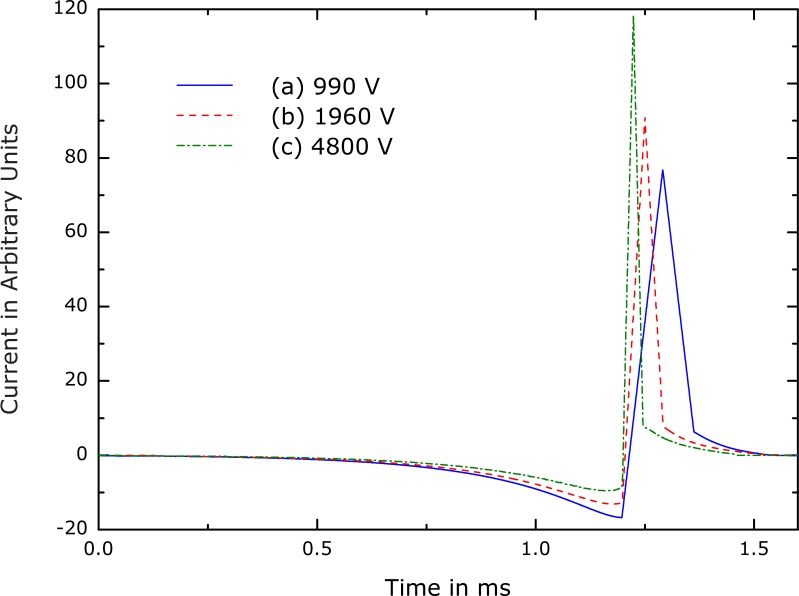
Waveform optima for various coil-voltage constraints. The waveforms correspond to the optima labeled (a), (b), and (c) in Figure 1. The corresponding relative coil voltages in arbitrary units are given in the legend, and the loss values obtained from Figure 1 are 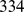, 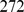, and 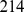, respectively. The comparison reveals the different roles of the individual pulse phases. Whereas the length of the second phase scales approximately inversely with the voltage constraint, the duration of the first phase remains constant. Nevertheless, the first phase amplitude varies relative to the second phase amplitude.

After the pre-evaluation of the waveform space, a refined optimization was performed for several voltages in the range of those typical in conventional devices. The number of degrees of freedom reached up to one thousand during these optimization runs. Thus, frequency components up to several hundred kilohertz could be accurately represented. The representative pulses labeled (a)–(c) in Figure 1 are plotted in Figures 2 and 3.

The best solutions are very similar to one another. The current always exhibits three relatively distinct phases. A slow first phase precedes a short large-amplitude second phase with triangular shape in the opposite direction. A separate third phase becomes visible after a large number of optimization iterations.

The corresponding coil voltage waveform (see Figure 2) is dominated by a biphasic rectangular pulse that has equal positive and negative amplitudes, corresponding to the rising and falling slopes of the second current phase, respectively. The small slopes of the first and third current phases make the corresponding voltage phases virtually indistinguishable from baseline.

The rectangularly shaped voltage during the second phase is likely a consequence of the second constraint, i.e., the voltage limit. The system uses the maximum allowed voltage level for the total duration of the second phase. This explains the positive swing of the voltage. The subsequent negative swing reduces the current as quickly as possible after the peak. Although this hyperpolarizing field might counteract excitation, a lower current level appears to be more important due to the dependence of the optimization objective on the squared coil current. The negative voltage is maintained at its maximum value until a certain low current level is reached from which the third phase converges to zero in a slow, approximately exponential decay.

Such reasoning can also be used to explain the slow negative first current phase. The first phase of the coil current introduces a negative bias for the onset of the second, depolarizing phase. While keeping the rise time constant, the peak current of the second phase is reduced by the magnitude of the shifted baseline at the end of the first phase. Thus, the negative bias created by the first phase decreases the energy loss by lowering the peak current in the second phase without reducing the pulse duration. The first phase contributes to the loss, of course, but its current amplitude is relatively low. Since the loss depends on the squared current, the first phase saves more energy due the reduced current in the second phase than the additional loss it contributes itself.

Importantly, the slow first phase of the coil current has a minimal effect on the neural dynamics. Accordingly, it biases the current and reduces the energy loss without significantly disrupting the membrane state. The weak influence of the first current phase on the state of the neuron can be demonstrated by inspecting the state of the modeled ion channels and the membrane potential. By the end of the first phase, the membrane potential has changed by less than 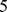
% for any of the three refined solutions from Figure 3. Accordingly, the dynamics of the single channel types (represented by the variables 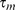, 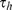, 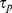, and 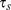) are practically unchanged. The state variables of the potassium and slow sodium channels, 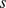 and 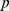, change by less than 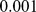
% and 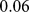
%, respectively. The change in the fast sodium channels is also small, with the activation and inactivation state variables, 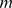 and 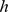, both changing by less than 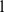
%. Changes of this order are too small to significantly affect the pulse development during the subsequent depolarizing phase of the pulse. Thus, the first phase of the coil current is optimized to reduce the electrical loss of the second, depolarizing phase, while minimally affecting the state of the neural membrane.

It should be noted that such an explanation of the mechanism of single phases is always a simplification. Ascribing certain functions to isolated parts of a pulse could be misleading in the context of nonlinear dynamics. Nevertheless, it is encouraging that the features of the optimal pulse shape are qualitatively consistent with simplified considerations of membrane dynamics in combination with electromagnetic induction.

### Dependence of the Optimal Waveform Features on Pulse Duration

The similarity among the waveforms in Figure 3 suggests that they can be treated as representatives of the same class. However, in contrast to sinusoidal or triangular current pulses, these optimal waveforms are not related by a simple linear transformation such as temporal scaling. The behavior of the first phase, which decreases if the amplitude of the second phase is increased, argues against such a simplification. If the second phase is isolated, however, a simple time scaling can be assumed in the first approximation. The pulse duration determines the required coil voltage level and influences the heating: For shorter triangles, the amplitude has to be increased, but the losses diminish. The dynamics of the first phase, however, do not change at all for the studied range, but only the amplitude is adjusted for the various voltage constraints.

**Figure 4:**
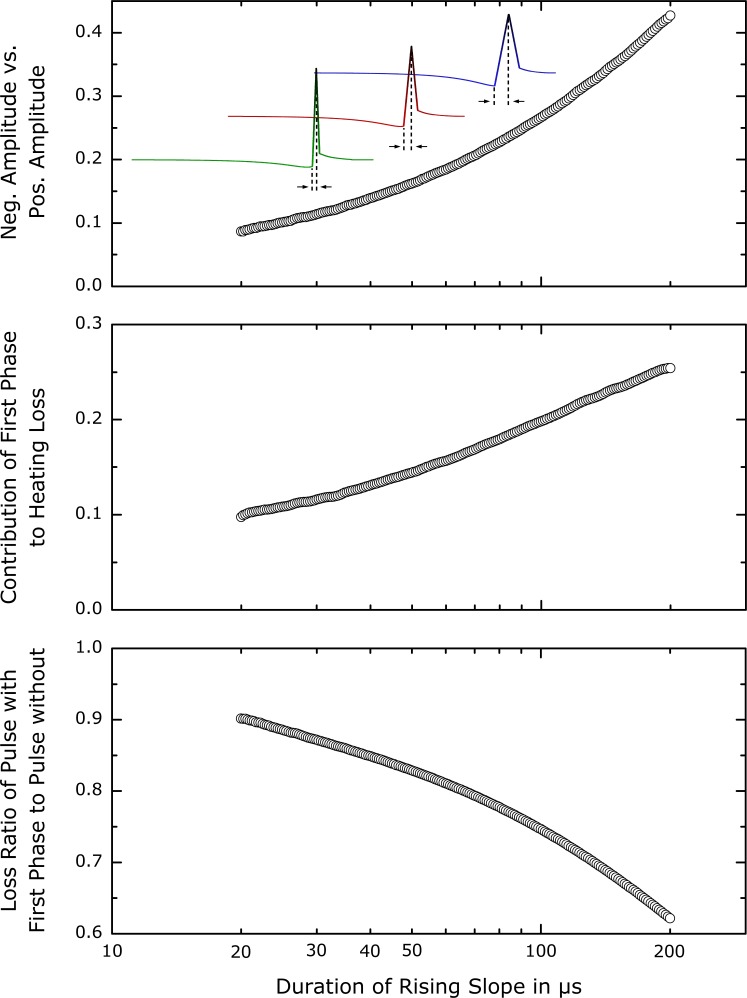
Role of the first phase in the optimal waveforms. The ratio of the negative current amplitude to the subsequent positive peak is shown in the top plot. The x-axis gives the length of the rising current slope (see inlay) which is equal to the duration of the positive voltage phase. As the pulse duration is increased for lower maximum coil voltage, the amplitude of the first phase rises relative to that of the second phase. The contribution of the first phase to ohmic losses (middle plot) is not constant, but increases with pulse duration. Although it amounts to a substantial portion of the total loss, the first phase is advantageous especially for long pulses as the bottom plot demonstrates. The waveform incorporating the first phase produces approximately two thirds of the energy loss at rise times around 200 µs compared to the same pulse without the first phase, both evaluated at their individual excitation threshold.

The detailed behavior of the optimal initial first phase was analyzed in a separate parameter study. The amplitude of the first phase, i.e., the value of the most negative point in the current profile, was set proportional to the main phase peak amplitude by an adjustable ratio. As a second degree of freedom, the duration of the rising slope of the main phase was varied; the falling slope changes proportionally to the rising slope. The optimal amplitude of the first phase was evaluated for various different main phase durations. The results are presented in Figure 4.

The top panel of Figure 4 shows the ratio of the first phase amplitude to the second phase amplitude. The middle panel displays the contribution of the first phase to the objective calculated as the ratio between the integral of the squared current over the first phase to the integral over the whole waveform. For very short pulses, the first phase nearly vanishes and the pulse becomes approximately triangular, corresponding to a biphasic rectangular voltage pulse. For brief pulses, the first phase contributes relatively little to the overall energy loss. On the other hand, for the longest depicted durations of the second phase the initial phase amplitude is almost half of the main amplitude and contributes a quarter of the energy loss.

Despite the substantial ohmic cost of the first phase, this investment is rewarding with respect to the total energy loss at the specific threshold. The bottom panel in Figure 4 visualizes the ratio of the losses of the optimized class of waveforms to the corresponding value without the first phase, i.e., an approximately triangular current. As the pulse duration increases, the advantage of the optimized waveforms over the simpler triangular current pulses grows. For a rise time of the second phase of 200 s, the loss of the best local minimum amounts to 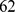
% of that for the corresponding triangular current pulse without a first phase. Again, for very short pulses, the loss ratio tends to one since the differences in shape between the two pulse types diminish as well.

### Generalizability

To explore the robustness of the optima with respect to the neuron model characteristics, we carried out optimization using two simpler models: the Motz-Rattay model [57] and the linear leaky integrate-and-fire model [19]. The Motz-Rattay model has dynamics that are markedly different from those of human motor axons. The leaky integrate-and-fire model is a regression model that is reduced to the absolute minimum requirements. Despite its limitations, this linear first-order neuron description is popular in magnetic stimulation because its single parameter–the so-called membrane time constant–can be extracted from experimental data [4], [55], [58]. For details on the neuron models, see the methods section.

**Figure 5:**
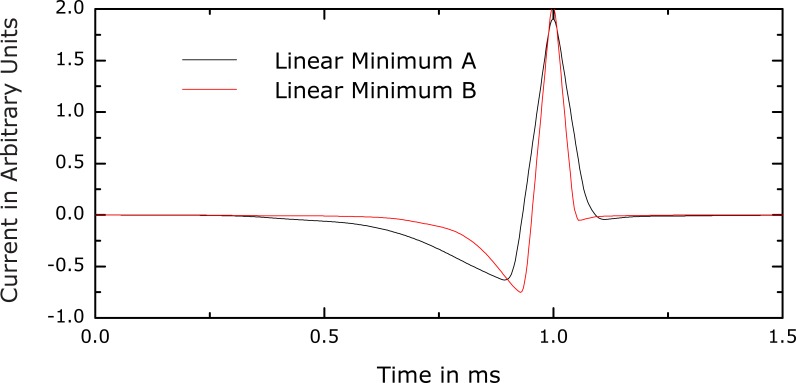
Optima for two different peak voltage levels for a simple linear first-order integrate-and-fire model with a time constant of 167 µs. Even in this extremely simplified model, the key pulse shape features obtained with the nominal neuron model (see Figure 3) are prominent, including the low amplitude, negative first phase and the triangular second phase with larger amplitude and equal rise and fall slope magnitudes. These examples further support the generalizability of the main optimization results.

**Figure 6:**
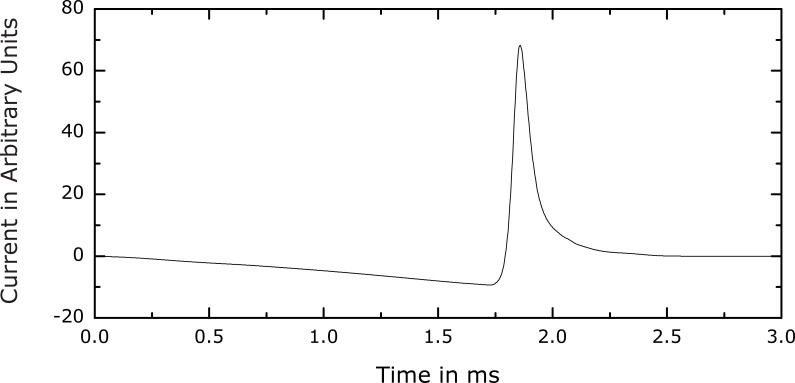
Best local minimum from an alternative axon model (Motz-Rattay). The characteristic phases observed in the more realistic model in the previous figures also appear here, supporting the robustness of the results.

Representative optimal current waveforms for the Motz-Rattay and integrate-and-fire models are shown in Figures 5 and 6, respectively. Figure 5 depicts two optima corresponding to two different values of the coil voltage constraint. Clearly, these optimal pulse waveforms display the characteristic pulse phases observed with our nominal model, despite the very different model types. All optimal pulse shapes have a negative current phase that precedes and has longer duration as well as lower amplitude than the subsequent positive current phase. Furthermore, the positive current phase is nearly triangular, with comparable rising and falling slopes. The similarity of the key features of the optimal pulses across this diverse set of neuron models supports the validity of our nominal results and their robustness with respect to neuronal type.

## Discussion

We implemented numerical optimization of the pulse shape for inductive stimulation of an axon with the objective of minimizing energy loss. The latter was quantified by the integral of the squared coil current. The optimization constraints were reduced to a minimum, comprising only a limit on the maximum coil voltage and the requirement that the axon model generates an action potential. Thus, the optimization was not limited to specific device circuitry or parametrization of predetermined waveforms, but rather was a practically unconstrained search of the pulse shape space.

The results were consistent across various initial conditions, voltage constraints, and alternative neuron models (see Figures 1, 2, 3, 5, and 6). The optimal pulse shapes consist of an initial slowly falling negative first current phase followed by a rapidly rising and falling second current phase, trailed by a slow decay to zero. The structure of these pulses is intuitively reasonable from the perspective of reducing the energy loss, as discussed in Section *Refined Optimization*. Even though intuitive explanations of phenomena involving nonlinear dynamics could be misleading, it is encouraging that the features of the optimal pulse shape are qualitatively consistent with simplified considerations of membrane dynamics in combination with electromagnetic induction.

The robustness of the solution is furthermore important for the question of generalizability. While the substantial similarity of motor axons with fibers originating from cortical pyramidal neurons in terms of channel expression [38], [39] suggests the applicability to this type, the high insensitivity with respect to specific neuron properties supports an applicability also for most other neural targets.

Especially, the linear model supports a general character of the results. First, this model reduces the dynamics to the common ground which almost all axons in the central and the peripheral nervous system share. Second, it is a regression of the whole black-box system using the coil current as its input [55]. Accordingly, also the three mechanistic uncertainties in cortical magnetic stimulation–i.e., the site of cortical stimulation, the recruited neuron types, and the activated segment in these neurons–are subsumed in this model by black-box regression.

The pulse shapes derived in this study cannot be generated by existing magnetic stimulation systems. Commercial devices generate exclusively sinusoidal pulses based on oscillator circuits and provide only limited control over pulse dynamics. Recently, a more flexible device that generates near-triangular current pulses with several degrees of control over the pulse parameters was presented [17]. Although the described embodiment was designed for relatively low negative voltages, which leads to asymmetric pulses, the underlying circuit topology could, in principle, produce also almost symmetric triangular pulses that notably outperform existing commercial devices. Alternatively, a variation of this circuit in form of a full-bridge pulse circuit with two individual capacitors with small differential voltage for each of the two half-brigdge subcircuits [59] could generate pulses resembling the optimal pulse shape. The difference of these two capacitors could be used for generating the slow first phase. This approach, however, would require large capacitors. Finally, a more flexible pulse shaping technology that allows the generation of virtually any waveform could accurately reproduce the optimal pulses derived here [60].

For the optimal pulse shapes, higher coil voltage results in lower energy loss, analogously to conventional magnetic stimulation waveforms (see Figure 1). In standard magnetic stimulation paradigms, the pulse amplitude is used to control the strength of stimulation. This is a natural choice since most conventional devices do not allow pulse-width adjustment due to the inflexible resonant circuit. Considering efficiency, however, an alternative approach for controlling the stimulation strength of a pulse may be advantageous. In devices with adjustable waveforms, the pulse loss can be reduced by using the highest available voltage level of a device and modulating the stimulation strength via the pulse width instead of the amplitude, especially in high-power applications [2]. However, for paradigms where the pulse dynamics are important for the stimulation outcome, such as selective stimulation of neural populations, diagnostics, or neural dynamics analysis, the conventional amplitude adjustment could be advantageous.

There are, however, practical limits to the use of extremely high voltages, even though these are in principle more efficient. These limits are driven by insulation considerations, semiconductor voltage ratings, high-frequency coil losses, and switching losses due to circuit capacitance. Such practical aspects are highly dependent on the implementation. The pulse parameters for specific circuit topologies could be optimized with the described approach by imposing additional constraints on the waveform shape.

The results of this study were obtained in a systematic way and show very plausible features. The outcome was not biased by initialization with current or voltage waveforms that anticipate the final results. Instead, the waveforms evolved solely based on the optimization objective and the constraints. The obtained shapes are relatively stable and reemerged in independent runs with random initialization of the parameters. Indeed, these solutions can be global or not far from the global solution.

Still, the optimized waveforms are the best found local minima of the problem formulation, which in turn is based on a nonlinear axon model. Each model has limitations and only approximates reality. Especially in the absolute task of minimization, a physiologic optimum could look slightly different, and could even be an individual characteristic of a specific neuron. Nevertheless, given the robustness of the optimum pulse shape features with respect to the neuron model, it is likely that these features would also be present in pulses optimized in vivo. As with any model-based approach, the numerical results should be considered as a starting point for experimental studies.

Finally, it should be noted that improving the energy efficiency is critical to functional and portable magnetic stimulation systems. The former can have very high power consumption, whereas the latter have limited battery energy. On the other hand, in some applications of magnetic stimulation, it may be more appropriate to improve the pulse shape to ensure best selectivity of stimulation of specific neural populations and/or to increase a repetitive stimulation protocol’s neuromodulation potency. For instance, the neural selectivity of a pulse could potentially be controlled by the relative amplitude and duration of the various pulse phases [14], [51]. Selective stimulation–which cannot be expected to be energy efficient–leverages on differences between distinct neural populations. As a consequence and in sharp contrast to the robustness of the minimum-energy waveforms with respect to the neuron, the waveforms obtained with such optimization might be highly sensitive to the specific characteristics of the target and nontarget neurons. Whereas the basic pulse features for minimum energy loss arose robustly for several kinds of linear and nonlinear low-pass dynamics, high selectivity is almost independent from the physical principles outside the neuron as long as their constraint of dc-free waveforms is met. Provided appropriately formulated objectives, the presented optimization framework, which allows nearly unconstrained waveform representation and optimization, is well suited for such alternative optimization goals.

## Methods

### Optimization Framework

**Figure 7:**
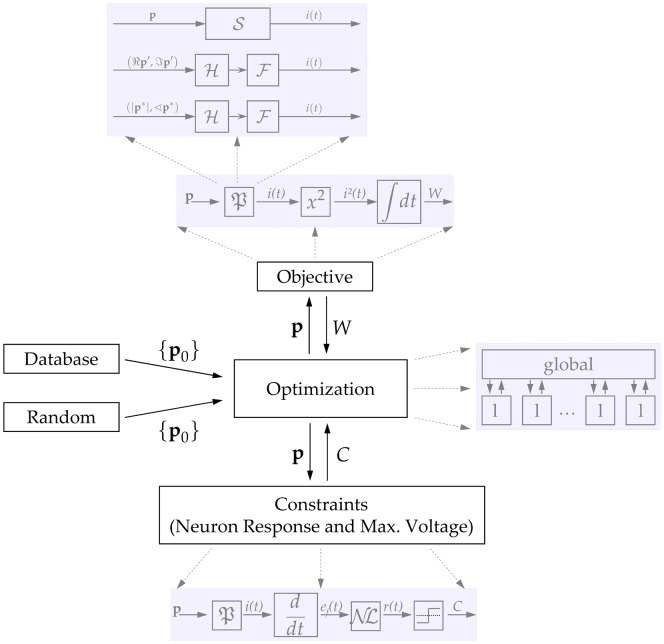
Structure of the optimization setup. The three basic blocks are further refined in grey. The minimization system has a hybrid structure. A global optimizer commands many local workers (l). The algorithms minimize the objective function 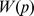 in the space of valid constraints 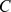, but have only access to a finite number of parameters 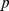. The latter are converted to the current function 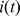 by 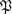 which is implemented with three alternative methods including spline generator functional 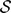 and Hilbert transform 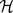 coupled with Fourier series 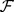 using either Cartesian (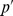) or polar form (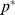). The initialization (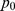) is performed with either random noise, conventional sinusoidal waveforms, or results from earlier runs (database). The axon model is incorporated into the constraints as a nonlinear element (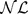) which processes the electric field waveform 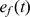 and returns a response 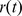.

The optimization framework has to describe the temporal dynamics of magnetic stimulation including all stages from the coil current to the neural response. The structure of the optimization setup is depicted in Figure 7 and discussed below.

Magnetic stimulation induces an electric field that is proportional to the coil current rate of change which, in turn, is proportional to the coil voltage [36]. It is assumed that the tissue around the axons does not filter nor distort the electric field, so the excitation current injected in the neural membrane is proportional to the first derivative of the coil current [36]. The neuron model hands back the first constraint (generation of action potential). The second constraint (limited coil voltage) and the objective functional (energy loss) were generated from the coil voltage 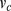 and current 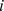, respectively.

The framework was implemented in C and optimized for maximum speed. For the solution of the differential equations, a standard second-order Runge-Kutta method was used. The allowed maximum time step for the final analysis was set to 500 ns. The whole optimization framework implementation followed a strict parallel design. Every call of the differential-equation solver was executed in a separate thread. The computation was performed on three eight-core Xeon servers for the low-dimensional problems and on a high-performance system of the Leibniz Supercomputing Centre of the Bavarian Academy of Sciences and Humanities.

### Waveform Parametrization

The objective and all constraints were formulated as functionals; the optimization task is a variational problem. For numerical handling, however, this abstract construction has to be translated into a finite set of numbers. Thus, an appropriate parametrization is an essential element, which moreover influences the stability of the optimization system. This mapping from the finite parameter space to a sufficient (not continuous, but dense in the mathematical sense) subspace of the Banach space of all waveforms allows a stable evaluation of all needed operators, such as derivatives, in an analytical way. Evading the issue of parametrization by taking a large number of sample points which are handed over to an optimization algorithm may risk instabilities in the context of inductive stimulation.

Various waveforms that do not share clear common features are able to stimulate a nerve. Thus, the objective function (energy loss) forms a surface in the waveform parameter space that has numerous local minima. Electromagnetic induction with its differentiating behavior further complicates the waveform optimization problem. The selection of an appropriate coordinate system for the waveform description can remarkably improve the solution process, but the problem is nonlinear and has no natural discrete parametrization. Therefore, we used simultaneously three coordinate systems to parametrize the coil current as their combination stabilizes the numerical processing.

First, the waveform is described by cubic spline curves; their finite parameters 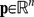 act as degrees of freedom for the optimization algorithm. This approach is similar to the core principles of the finite element method in numerics. The predefined solution forms a subspace of the class of all functions with continuous second derivative 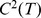, with 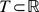 being the compact support, i.e., the limited time span of the nonzero waveform. This was set to three milliseconds during the piloting phase as no local minimum was observed to require a longer time base. At the boundaries of 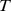, a Dirichlet condition forces the current to zero. However, in the context of inductive stimulation, this coordinate system still has a high density of local minima in the objective functional.

Therefore, parameter description in frequency space was added. A complex-valued parameter vector 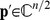 can be taken without further constraints. A discrete Hilbert transform provides the coefficients of a complex-valued Fourier series. The latter is a smooth function and a member of 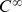, accordingly.

The optimization algorithm processes only plain one-dimensional floating-point numbers. There are two simple ways for generating the required complex-valued parameter vector from a real-valued vector 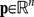. One uses a Cartesian representation with a real part 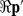 and an imaginary contribution 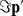; the other describes the complex plane in polar coordinates with magnitude 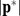 and phase 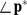. For the second method, the phase values are multiplied by a constant factor in order to match their range with the magnitude of the complex numbers in a typical parameter scaling step.

All of the three proposed methods of parametrization have at least two continuous derivatives. All of them allow a dynamic change of the coordinate system. This comprises a conversion from one parametrization to another, as well as increasing or reducing the degrees of freedom in the same type of description.

The dynamic change of coordinate systems addresses instability due to the vast number of local minima. A local minimum in one coordinate space might not be pronounced in another. Many observed local minima showed artifacts typical for one type of description, such as Gibbs phenomena or ringing, which are not stable for the optimizer with another parametrization. Furthermore, a dynamic change of the number of dimensions was used for adaptive control over stability as described below.

### Optimization Algorithm

We implemented a method for multivariate minimization of the parametrized objective. A global method was chosen due to the large number of local minima. For a quick convergence, the global method was combined with a local algorithm in a hybrid approach. Robust local optimization workers converge rapidly on dominant local minima. The global framework in turn combines the information about these local minima.

For the global algorithm, a particle swarm method was selected [61]. The term *particle* denotes a single waveform parameter set in this context. A particle swarm framework with many local workers allows a concurrent design that is well suited for high-performance computing.

Two algorithms, a simplex-derived constrained optimization by linear approximation (COBYLA) [62], [63] and an interior-point method [64], act alternatively as relatively stable local optimizers. The type of coordinate system of each worker is fixed. The number of degrees of freedom applies to all workers and is controlled by the global framework. The degrees of freedom are increased by a predefined step if convergence is achieved and the result outperforms the previous step; otherwise, the degrees of freedom are decreased.

The global method hands over the parameters to the local workers who detect a nearby valid local minimum that fulfills all constraints. Their results are considered in deciding every particle’s best as well as the total best in each step. In order to avoid oscillations in the attraction field of local minima, the position of a given particle is not changed to the result of the corresponding local worker.

The particle swarm algorithm updates the parameters 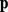 of the 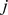-th particle in the 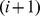-th step
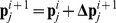
(2)

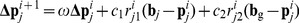
(3)where 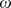 is the inertia, 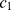 and 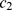 are the gravity parameters, and 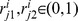 are the modulation variables corresponding to the local best and the global best, respectively [61]. The modulation variable values are chosen randomly in every step. The global best is denoted by 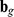, while each particle has its own local best 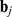. For the constants, several alternatives were tested, and the values 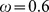, 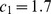, 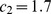 were chosen. Notably, the algorithm performed appropriately even with repellent behavior, which was used especially for exploration of the parameter space (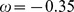, 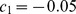, 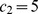). The number of particles was up to fifty. The particles were initialized with random numbers. In several runs, a fraction of the workers was initialized with conventional waveforms.

### Neuron Model

The decision if a pulse elicits an action potential is made by a nonlinear model of a human motor axon. The nominal model is a local axon description incorporating fast sodium, persistent sodium, and dominant slow potassium channels as well as passive leakage. The formulation and parameters of the membrane dynamics are based on the Schwarz model [65]–[67], likewise a local single-segment approach, and includes parameter updates by the same collaborators [68], [69]. as well as by McIntyre et al. [70]. All model parameters are given in Appendix S1. The first optimization constraint (action potential generation) was defined to be fulfilled if the neuron potential exceeded +10 mV after a stimulus.

Like other local or single-compartment models common in neurodynamics [24], [65], [71], [72], this neuron description does not incorporate signal propagation, but concentrates on the excitation dynamics. The electromagnetic induction process enforces an electric field across the membrane locally at a node. This is reflected by the typical activating function in electrical and magnetic stimulation which does not interact with the stimulation dynamics, but extracts the transmembrane component of the induced electric field differentially, keeping its time course [36], [73]. Accordingly, there is a consensus that external stimulation can be treated equally to intracellular current injection [11], [74] as used in whole-cell patch-clamp measurements from which local neuron models directly derive.

To check the robustness of the waveform optima with respect to the neuron model parameters, we also optimized the pulse dynamics using two alternative models. We implemented the Motz-Rattay model and a simple leaky integrate-and-fire model. The Motz-Rattay model is based on a modification of the original Hodgkin-Huxley equations to account for mammalian body temperatures and provides a relatively simple and computationally fast approach for studying pulse dynamics, for example, of auditory nerve axons [57]. Due to its notable deviation in dynamics compared to human axons, it is not well suited as a nominal model in this study, but it can provide a level of validation for the main characteristics of the optimized waveforms.

The linear leaky integrate-and-fire model is widely used in computational neuroscience and is commonly deployed as a regression model in magnetic stimulation to fit experimental strength-duration data [4], [55], [58]. At present, this type of model is the only experimentally derived description of magnetic stimulation dynamics. It reduces the dynamical complexity to a minimum that almost all axons have in common, encompassing therefore the excitation of various types of targets including motor, cortico-spinal, or interneuron axons. However, nonlinearities–which are, for example, already important in case of brief pulses and for the explanation of phenomena such as the threshold reduction for multiphasic waveforms–are missing. Based on the range of neural membrane time constant values derived with magnetic stimulation of both cortical and peripheral neurons (116 µs –196 µs; see [4], [55], [58]), we chose a value of 167 µs here.

## Supporting Information

Appendix S1 **Details on the used nominal axon model.**
(PDF)
