# Supplementary material for: Analysis and Optimization of Pulse Dynamics for Magnetic Stimulation
Source: PLoS One. 2013 Mar 1;8(3):e55771. doi: 10.1371/journal.pone.0055771 (PMC3585867; doi:10.1371/journal.pone.0055771)
Supplement: Appendix S1 — Details on the used nominal axon model. (PDF) [file pone.0055771.s001.pdf]

Appendix

The nerve model uses a local, differential formalism which describes the excitation process without propagation. The model structure and parameter values are based on (65; 66; 67) with updates from (68; 69; 70), and are given below. The first element of the state vector ϕ denotes the membrane potential v; the remaining elements describe the channel dynamics m, h, p, s which correspond to the states of first-order systems with nonlinear time constants. Variable e_f denotes the excitation function, which is directly proportional to the electric field waveform.

$$\phi = \begin{pmatrix} \phi_1 \\ \phi_2 \\ \phi_3 \\ \phi_4 \\ \phi_5 \end{pmatrix} \equiv \begin{pmatrix} v \\ m \\ h \\ p \\ s \end{pmatrix}$$

$$\frac{\partial}{\partial t}\phi = \begin{pmatrix} \frac{1}{c_m}\left(e_f(t) + \left(g_{Na}\phi_2^3\phi_3 + g_{Na,p}\phi_4^3\right)(V - E_{Na}) - g_K\phi_5(V - E_K) - g_l(V - E_l)\right) \\ \frac{m_\infty - \phi_2}{\tau_m(\phi_1)} \\ \frac{h_\infty - \phi_3}{\tau_h(\phi_1)} \\ \frac{p_\infty - \phi_4}{\tau_p(\phi_1)} \\ \frac{s_\infty - \phi_5}{\tau_s(\phi_1)} \end{pmatrix}$$

$c_m = 2.0\,\mu\text{F/cm}^2$

$g_{Na} = 290\,\text{mS/cm}^2$
$g_{Na,p} = 25\,\text{mS/cm}^2$
$g_K = 80\,\text{mS/cm}^2$
$g_l = 7\,\text{mS/cm}^2$
$E_{Na} = 50.0\,\text{mV}$
$E_K = -90.0\,\text{mV}$
$E_l = -90.0\,\text{mV}$
$T = 310\,\text{K}$

$\delta_m = 1.86\,(\text{mV ms})^{-1}$
$\epsilon_m = 21.4\,\text{mV}$
$\zeta_m = 10.3\,\text{mV}$
$\eta_m = 0.086\,(\text{mV ms})^{-1}$
$\theta_m = 25.7\,\text{mV}$
$\iota_m = 9.16\,\text{mV}$
$\delta_h = 0.062\,(\text{mV ms})^{-1}$
$\epsilon_h = 114.0\,\text{mV}$
$\zeta_h = 11.0\,\text{mV}$
$\eta_h = 2.3\,\text{ms}^{-1}$
$\theta_h = 31.8\,\text{mV}$
$\iota_h = 13.4\,\text{mV}$
$\delta_p = 0.01\,(\text{mV ms})^{-1}$
$\epsilon_p = 27\,\text{mV}$

$\zeta_p = 10.2\,\text{mV}$
$\eta_p = 2.5 \cdot 10^{-4}\,(\text{mV ms})^{-1}$
$\theta_p = 34\,\text{mV}$
$\iota_p = 10\,\text{mV}$
$\delta_s = 0.3\,\text{ms}^{-1}$
$\epsilon_s = 53\,\text{mV}$
$\zeta_s = -5\,\text{mV}$
$\eta_s = 0.03\,\text{ms}^{-1}$
$\theta_s = 90\,\text{mV}$
$\iota_s = -1\,\text{mV}$

$T_{r,act} = 293\,\text{K}$
$T_{r,dea} = 293\,\text{K}$
$T_{r,K} = 309\,\text{K}$

$\kappa_{act} = \exp\left(\frac{(T - T_{r,act})\ln 2.2}{10\,\text{K}}\right)$
$\kappa_{dea} = \exp\left(\frac{(T - T_{r,dea})\ln 2.9}{10\,\text{K}}\right)$
$\kappa_K = \exp\left(\frac{(T - T_{r,K})\ln 3.0}{10\,\text{K}}\right)$

$\alpha_m = \kappa_{act}\psi_{m\alpha}(\phi_1)$
$\beta_m = \kappa_{act}\psi_{m\beta}(\phi_1)$
$\tau_m = \frac{1}{\alpha_m + \beta_m}$
$m_\infty = \frac{\alpha_m}{\alpha_m + \beta_m}$

$$\alpha_h = \kappa_{dea}\psi_{h\alpha}(\phi_1)$$
$$\beta_h = \frac{\kappa_{dea}\eta_h}{1+\exp\left(-\frac{v+\theta_h}{\iota_h}\right)}$$
$$\tau_h = \frac{1}{\alpha_h+\beta_h}$$
$$h_\infty = \frac{\alpha_h}{(\alpha_h+\beta_h)}$$

$$\alpha_p = \kappa_{act}\psi_{p\alpha}(\phi_1)$$
$$\beta_p = \kappa_{act}\psi_{p\beta}(\phi_1)$$
$$\tau_p = \frac{1}{\alpha_p+\beta_p}$$
$$p_\infty = \frac{\alpha_p}{\alpha_p+\beta_p}$$

$$\alpha_s = \frac{\kappa_K\delta_s}{1+\exp\left(\frac{\phi_1+\epsilon_s}{\zeta_s}\right)}$$
$$\beta_s = \frac{\kappa_K\eta_s}{1+\exp\left(\frac{\phi_1+\theta_s}{\iota_s}\right)}$$

$$\tau_s = \frac{1}{\alpha_s+\beta_s}$$

$$s_\infty = \frac{\alpha_s}{\alpha_s+\beta_s}$$

$$\psi_{m\alpha} = \begin{cases} \delta_m\zeta_m & \text{for } \phi_1 = -\epsilon_m \\ \frac{\delta_m(\phi_1+\epsilon_m)}{1-\exp\left(-\frac{\phi_1+\epsilon_m}{\zeta_m}\right)} & \text{else} \end{cases}$$

$$\psi_{m\beta} = \begin{cases} -\eta_m\iota_m & \text{for } \phi_1 = -\theta_m \\ -\frac{\eta_m(\phi_1+\theta_m)}{1-\exp\left(\frac{\phi_1+\theta_m}{\iota_m}\right)} & \text{else} \end{cases}$$

$$\psi_{h\alpha} = \begin{cases} -\delta_h\zeta_h & \text{for } \phi_1 = -\epsilon_h \\ -\frac{\delta_h(\phi_1+\epsilon_h)}{1-\exp\left(\frac{\phi_1+\epsilon_h}{\zeta_h}\right)} & \text{else} \end{cases}$$

$$\psi_{p\alpha} = \begin{cases} \delta_p\zeta_p & \text{for } \phi_1 = -\epsilon_p \\ \frac{\delta_p(\phi_1+\epsilon_p)}{1-\exp\left(-\frac{\phi_1+\epsilon_p}{\zeta_p}\right)} & \text{else} \end{cases}$$

$$\psi_{p\beta} = \begin{cases} -\eta_p\iota_p & \text{for } \phi_1 = -\theta_p \\ -\frac{\eta_p(\phi_1+\theta_p)}{1-\exp\left(\frac{\phi_1+\theta_p}{\iota_p}\right)} & \text{else} \end{cases}$$

References

1. Weber M, Eisen A (2002) Magnetic stimulation of the central and peripheral nervous systems. *Muscle & Nerve*, 25(2):160–175.

2. Szecsi J, Götz S, Pöllmann W, Straube A (2010) Force-pain relationship in functional magnetic and electrical stimulation of subjects with paresis and preserved sensation. *Clinical Neurophysiology*, 121(9):1589–1597.

3. Hsu K-H, Nagarajan SS, Durand DM (2003) Analysis of efficiency of magnetic stimulation. *IEEE Transactions on Biomedical Engineering*, 50(11):1276–1285.

4. Barker AT, Garnham CW, Freeston IL (1991) Magnetic nerve stimulation: the effect of waveform on efficiency, determination of neural membrane time constants and the measurement of stimulator output. *Electroencephalography and Clinical Neurophysiology Supplement*, 43:227–237.

5. Ueno S, Tashiro T, Harada K (1988) Localized stimulation of neural tissue in the brain by means of a paired configuration of time-varying magnetic fields. *Journal of Applied Physics*, 64(10):5862–5864.

6. Ruohonen J, Virtanen J, Ilmoniemi RJ (1997) Coil optimization for magnetic brain stimulation. *Annals of Biomedical Engineering*, 25(5):840–849.

7. Salinas FS, Lancaster JL, Fox PT (2007) Detailed 3D models of the induced electric field of transcranial magnetic stimulation coils. *Physics in Medicine and Biology*, 52(10):2879-2892.

8. Cadwell J (1991) Optimizing magnetic stimulator design. *Electroencephalography and Clinical Neurophysiology Supplement*, 43:238–248.

9. Goetz SM, Herzog HG, Gattinger N, Gleich B (2011) Comparison of coil designs for peripheral magnetic muscle stimulation. *Journal of Neural Engineering*, 8(5):056007.

10. Lorenzen HW, Weyh T (1992) Practical application of the summation method for 3-d static magnetic field calculation of a setup of conductive and ferromagnetic material. *IEEE Transactions on Magnetics*, 28(2):1481–1884.

11. Davey KR, Epstein CM (2000) Magnetic stimulation coil and circuit design. *IEEE Transactions on Biomedical Engineering*, 47(11):1493–1499.

12. Polson MJR, Barker AT, Freeston IL (1982) Stimulation of nerve trunks with time-varying magnetic fields. *Medical and Biological Engineering*, 20(2):243–244.

13. Weyh T (1995) Magnetstimulation neuronaler Systeme. PhD thesis, UAF Munich.

14. Niehaus L, Meyer B-U, Weyh T (2000) Influence of pulse configuration and direction of coil current on excitatory effects of magnetic motor cortex and nerve stimulation. *Clinical Neurophysiology*, 111(1):75–80.

15. Sommer M, Alfaro A, Rummel M, Speck S, Lang N, et al. (2006) Half sine, monophasic and biphasic transcranial magnetic stimulation of the human motor cortex. *Clinical Neurophysiology*, 117(4):838–844.

16. Kammer T, Beck S, Thielscher A, Laubis-Herrmann U, Topka H (2001) Motor threshold in humans: a transcranial magnetic stimulation study comparing different pulse waveforms, current directions and stimulator types. *Clinical Neurophysiology*, 112:250–258.

17. Peterchev AV, Murphy DL, Lisanby SH (2011) Repetitive transcranial magnetic stimulator with controllable pulse parameters. *Journal of Neural Engineering*, 8(3):036016.

18. Havel WJ, Nyenhuis JA, Bourland JD, Foster KS, Geddes LA, et al. (1997) Comparison of rectangular and damped sinusoidal dB/dt waveforms in magnetic stimulation. *IEEE Transactions on Magnetics*, 33(5):4269–4271.

19. Lapicque L (1907) Recherches quantatives sur l'excitation électrique des nerfs traitée comme une polarisation. *Journal de Physiologie et de Pathologie Générale*, 9:620–635.

20. Offner F (1946) Stimulation with minimum power. *Journal of Neurophysiology*, 9:387–390.

21. Jezernik S, Sinkjaer T, Morari M (2010) Charge and energy minimization in electrical/magnetic stimulation of nervous tissue. *Journal of Neural Engineering*, 52(4):740–743.

22. Foutz TJ, McIntyre CC (2010) Evaluation of novel stimulus waveforms for deep brain stimulation. *Journal of Neural Engineering*, 7(6):066008.

23. Wongsarnpigoon A, Woock JP, Grill WM (2010) Efficiency analysis of waveform shape for electrical excitation of nerve fibers. *IEEE Transactions on Neural Systems and Rehabilitation Engineering*, 18(3):319–328.

24. Grill WM, Mortimer JT (1997) Inversion of the current-distance relationship by transient depolarization. *IEEE Transactions on Biomedical Engineering*, 44(1):1–9.

25. Dean D, Lawrence PD (1985) Optimization of neural stimuli based upon a variable threshold potential. *IEEE Transactions on Biomedical Engineering*, 32(1):8–14.

26. Gorman PH, Mortimer JT (1983) The effect of stimulus parameters on the recruitment characteristics of direct nerve stimulation. *IEEE Transactions on Biomedical Engineering*, 30(7):407–414.

27. Bütikofer R, Lawrence PD (1978) Electrocutaneous nerve stimulation—I: Model and experiment. *IEEE Transactions on Biomedical Engineering*, 25(6):526–531.

28. Bütikofer R, Lawrence PD (1979) Electrocutaneous nerve stimulation—II: Stimulus waveform selection. *IEEE Transactions on Biomedical Engineering*, 26(2):69–75.

29. Wongsarnpigoon A, Grill WM (2010) Energy-efficient waveform shapes for neural stimulation revealed with a genetic algorithm. *Journal of Neural Engineering*, 7(4):046009.

30. Claus D, Murray NMF, Spitzer A, Flügel D (1990) The influence of stimulus type on the magnetic excitation of nerve structures. *Electroencephalography and Clinical Neurophysiology*, 75(4):342–349.

31. Hiwaki O, Ueno S (1991). Experimental and modeling studies on properties of nerve excitation elicited by magnetic stimulation. *IEEE EMBC*, 13(2):853–854.

32. Reilly JP (1989) Peripheral nerve stimulation by induced electric currents: exposure to time-varying magnetic fields. *Medical and Biological Engineering and Computing*, 27(2):101–110.

33. Nagarajan SS, Durand DM, Warman EN (1993) Effect of induced electric fields on finite neuronal structures: a simulation study. *IEEE Transactions on Biomedical Engineering*, 40(11):1175–1188.

34. Maccabee PJ, Nagarajan SS, Amassian VE, Durand DM, Szabo AZ, et al. (1998) Influence of pulse sequence, polarity and amplitude on magnetic stimulation of human and porcine peripheral nerve. *Journal of Physiology*, 513(2):571–585.

35. Weyh T, Wendicke K, Mentschel C, Zantow H, Siebner H (2005) Marked differences in the thermal characteristics of figure-of-eight shaped coils used for repetitive transcranial magnetic stimulation. *Journal of Clinical Neurophysiology*, 116(6):1477–1486.

36. Ruohonen J, Panizza M, Nilsson J, Ravazzani P, Grandori F, et al. (1996) Transverse-field activation mechanism in magnetic stimulation of peripheral nerves. *Electroencephalography and Clinical Neurophysiology*, 101(2):167–174.

37. Machetanz J, Bischoff C, Pichlmeier R, Riescher H, Meyer B-U, et al. (1994) Magnetically induced muscle contraction is caused by motor nerve stimulation and not by direct muscle activation. *Muscle & Nerve*, 17:1170–1175.

38. Caldwell JH, Schaller KL, Lasher RS, Peles E, Levinson SR (2000) Sodium channel Nav1.6 is localized at nodes of Ranvier, dendrites, and synapses. *Proceedings of the National Academy of Sciences*, 97(10):5616–5620.

39. Tzoumaka E, Tischler AC, Sangameswaran L, Eglen RM, Hunter JC, et al. (2000) Differential distribution of the tetrodotoxin-sensitive rPN4/NaCh6/Scn8a sodium channel in the nervous system. *Journal of Neuroscience Research*, 60:37–44.

40. Skinner F, Sagara F (2010) Single neuron models: Interneurons. In: V. Vutsuridis et al. editors. Hippocampal Microcircuits, Springer, Berlin/New York, pp. 399–422.

41. Goldin AL (1999) Diversity of mammalian voltage-gated sodium channels. *Annals of the New York Academy of Sciences*, 868:38–50.

42. Whitaker WRJ, Faull RLM, Waldvogel HJ, Plumpton CJ, Emson PC, et al. (2001) Comparative distribution of voltage-gated sodium channel proteins in human brain. *Molecular Brain Research*, 88:37–53.

43. Birdno MJ, Kuncel AM, Dorval AD, Turner DA, Gross RE, et al. (2012) Stimulus features underlying reduced tremor suppression with temporally patterned deep brain stimulation. *Journal of Neurophysiology*, 107:364–383.

44. Buhlmann J, Hofmann L, Tass PA, Hauptmann C (2011) Modeling of a segmented electrode for desynchronizing deep brain stimulation. *Frontiers in Neuroengineering.* 4(15):1–8.

45. Grill WM, Cantrell MB, Robertson MS (2008) Antidromic propagation of action potentials in branched axons: Implications for the mechanism of action of deep brain stimulation. *Journal of Computational Neuroscience*, 24:81–93.

46. Butson CR, Cooper SE, Henderson JM, McIntyre CC (2007) Patient-specific analysis of the volume of tissue activated during deep brain stimulation. *Neuroimage*, 34(2):661–670.

47. Sotiropoulos SN, Steinmetz PN (2007) Assessing the direct effects of deep brain stimulation using embedded axon models. *Journal of Neural Engineering*, 4:107–119.

48. Ni Z, Charab S, Gunraj C, Nelson AJ, Udupa K, et al. (2011) Transcranial magnetic stimulation in different current directions activates separate cortical circuits. *Journal of Neurophysiology*, 105:749–756.

49. Di Lazzaro V, Oliviero A, Provice P, Meglio M, Cioni B, et al. (2001) Descending spinal cord volleys evoked by transcranial magnetic and electrical stimulation of the motor cortex leg area in conscious humans. *Journal of Physiology*, 537(3):1047–1058.

50. Pitcher JB, Ogston KM, Miles TS (2003) Age and sex differences in human motor cortex input-output characteristics. *The Journal of Physiology (London)*, 546(2):605–613.

51. Taylor JL, Loo CK (2007) Stimulus waveform influences the efficacy of repetitive transcranial magnetic stimulation. *Journal of Affective Disorders*, 97(1–3):271–276.

52. Öberg PÅ (1973) Magnetic stimulation of nerve tissue. *Medical and Biological Engineering*, 11(1):55–64.

53. McRobbie D, Foster MA (1984) Thresholds for biological effects of time-varying magnetic fields. *Clinical Physics and Physiological Measurements*, 5(2):67–78.

54. McRobbie D (1985) Design and instrumentation of a magnetic nerve stimulator. *Journal of Physics E: Scientific Instruments*, 18(1):74–78.

55. Peterchev AV, Jalinous R, Lisanby SH (2008) A transcranial magnetic stimulator inducing near-rectangular pulses with controllable pulse width (cTMS). *IEEE Transactions on Biomedical Engineering*, 55(1):257–266.

56. Rushton WAH (1932) Lapicque's canonical strength duration curve. *The Journal of Physiology*, 74(4):424–440.

57. Motz H, Rattay F (1986) A study of the application of the Hodgkin-Huxley and the Frankenhaeuser-Huxley model for electrostimulation of the acoustic nerve. *Neuroscience*, 18(3):699–712.

58. Peterchev AV, Westin G, Luber B, Lisanby S (2011) Corticospinal response characterization with controllable pulse parameter transcranial magnetic stimulation (cTMS). *Clinical Neurophysiology Supplement*, 112:S191.

59. Peterchev AV (2011) Circuit topology comparison and design analysis for controllable pulse parameter transcranial magnetic stimulators. *Proceedings of the IEEE EMBS Neural Engineering Conference*, 646–649.

60. Goetz SM, Pfaeffl M, Huber J, Singer M, Marquardt R, et al. (2012) Circuit topology and control principle for a first magnetic stimulator with fully controllable waveform. *Proceedings of the Annual Conference of the IEEE EMBS*, 4700–4703.

61. Eberhart R, Kennedy J (1995) A new optimizer using particle swarm theory. *Proceedings of the Sixth International Symposium on Micro Machine and Human Science 1995*, 39–43.

62. Powell MJD (1994) A direct search optimization method that models the objective and constraint functions by linear interpolation. *Advances in Optimization and Numerical Analysis*, Kluwer Academic Press, 51–67.

63. Powell MJD (2003) On trust region methods for unconstrained minimization without derivatives. *Mathematical Programming*, 97(3):605–623.

64. Waltz RA, Morales JL, Nocedal J, Orban D (2006) An interior algorithm for nonlinear optimization that combines line search and trust region steps. *Mathematical Programming*, 107(3):391–408.

65. Schwarz JR, Reid G, Bostock H (1995) Action potentials and membrane currents in the human node of Ranvier. *Pflügers Archiv: European Journal of Physiology*, 430(2):283–292.

66. Scholz A, Reid G, Vogel W, Bostock H (1993) Ion channels in human axons. *Journal of Neurophysiology*, 70(3):1274–1279.

67. Safronov BV, Kampe K, Vogel W (1993) Single voltage-dependent potassium channels in rat peripheral nerve membrane. *Journal of Physiology*, 460:675–691.

68. Richardson AG, McIntyre CC, Grill WM (2000) Modelling the effects of electric fields of nerve fibres: Influence of the myelin sheath. *Medical & Biological Engineering & Computing*, 38:438-446.

69. Reid G, Scholz A, Bostock H, Vogel W (1999) Human axons contain at least five types of voltage-dependent potassium channel. *Journal of Physiology*, 518(3):681–696.

70. McIntyre CC, Richardson AG, Grill WM (2002) Modeling the excitability of mammalian nerve fibers: influence of afterpotentials on the recovery cycle. *Journal of Neurophysiology*, 87(2):995–1006.

71. Gerstner W, Naud R (2009) How good are neuron models? *Science*, 326:379–380.

72. Pospischil M, Toledo-Rodriguez M, Monier C, Piwkowska Z, Bal T, et al. (2008) Minimal Hodgkin-Huxley type models for different classes of cortical and thalamic neurons. *Biological Cybernetics*, 99:427–441.

73. Rattay F (1986) Analysis of models for external stimulation of axons. *IEEE Transactions on Biomedical Engineering*, 33(10):974–977.

74. Bostock H (1983) The strength-duration relationship for excitation of myelinated nerves: computed dependence on membrane properties. *Journal of Physiology*, 341:59–74.
